# Association between SARS-CoV-2 in wastewater and COVID-19 hospitalizations in three countries, 2022–2024

**DOI:** 10.3389/fpubh.2025.1679596

**Published:** 2025-12-18

**Authors:** Mustapha M. Mustapha, Laura E. Choi, Tobias Bergroth, Hannah R. Volkman, Kate Devlin, Jingyan Yang, Alon Yehoshua, Farid L. Khan, John M. McLaughlin, Jennifer L. Nguyen

**Affiliations:** 1 Pfizer Inc., New York, NY, United States; 2 Department of Population Health, Hofstra University, Hempstead, NY, United States; 3 The Institute for Social and Economic Research and Policy, Columbia University, New York, NY, United States

**Keywords:** COVID, hospitalization, vaccine (COVID-19), wastewater, SARS-CoV-2 infection (COVID-19)

## Abstract

**Importance:**

As fewer jurisdictions report national COVID-19 hospitalization rates and testing of mild and asymptomatic cases is reduced, there is a need to better understand the relationship between COVID-19 hospitalizations and alternative measures of COVID-19 circulation, such as wastewater surveillance.

**Objective:**

We described the association between levels of SARS-CoV-2 in wastewater and COVID-19 hospitalization rates at the national level during and after the pandemic and explored whether wastewater virus level can predict COVID-19 hospitalization rates.

**Design, setting, and participants:**

Retrospective analysis of public health reports of national wastewater surveillance and COVID-19 hospitalizations from Denmark, the Netherlands, and the United States from 2022 to 2024.

**Main outcomes and measures:**

For each country, we calculated Pearson correlation coefficients and hospitalization multipliers (defined as the hospitalization rate for a given scaled wastewater virus level), for the overall study period and by Omicron subvariant predominance. Additionally, we developed linear regression models using scaled wastewater virus levels to predict concurrent and projected (1–4 weeks) COVID-19 hospitalization rates.

**Results:**

There was a strong correlation between national SARS-CoV-2 wastewater virus levels and weekly new COVID-19 hospitalization rates per million (0.86 [95% CI, 0.82 to 0.90], 0.80 [95% CI, 0.72 to 0.85], and 0.89 [95% CI, 0.85 to 0.92] in Denmark, the Netherlands, and the United States, respectively). Correlations were consistently strong across all subvariant predominance periods in all three countries (range, 0.72 to 1.0). Results from linear regression models showed that hospitalization rate lagged wastewater SARS-CoV-2 levels by approximately 1 week. Linear regression models best predicted hospitalizations 1 week into the future (range of mean absolute percentage error, MAPE, 11.2 to 22.6%) with decreasing prediction accuracy within the range of 2–4 weeks (range of MAPE, 32.5 to 62.3% at 4 weeks). The median hospitalization multiplier (defined as ratio of weekly hospitalization rate to scaled wastewater SARS-CoV-2 level) were 859.3 (IQR, 621.7 to 1210.4), 178.3 (IQR, 133.7 to 243.7), and 245.9 (IQR, 184.0 to 293.7) for Denmark, the Netherlands, and the United States, respectively, during the study period. Regression models identified significant reduction in hospitalizations for a given wastewater virus level over time in all three countries.

**Conclusions and Relevance:**

SARS-CoV-2 wastewater virus levels were strongly correlated with COVID-19 hospitalization rates in the upcoming week. Wastewater to hospitalization ratios can be leveraged to enhance public health decision-making and resource allocation.

## Introduction

COVID-19 hospitalizations are an important measure of disease severity and an indicator of the public health burden of COVID-19 (1). National COVID-19 hospitalization rates reflect the overall strain on healthcare systems, guiding resource allocation and policy decisions including emergency preparedness (2, 3). Unlike case counts, which can be influenced by testing availability and individual reporting behaviors, hospitalization rates provide a more stable and clinically meaningful measure of disease burden. Monitoring hospitalization rates at the national level enables timely, coordinated responses to surges in severe illness and supports strategic planning for current and future public health threats (2, 3).

Following declaration of an end to the pandemic by the World Health Organization in May 2023, there was decreased reporting of SARS-CoV-2 infections and COVID-19 hospitalizations. This reduction in reporting in the post-pandemic period has increased the need for alternative measures of disease burden, such as wastewater SARS-CoV-2 levels (4). Wastewater surveillance has emerged as an important measure of community-level infection trends independent of the availability of COVID-19 testing, and can provide an early measure of potential increases in more severe illness (5–7). Such early data may aid timely public health decision-making and resource planning, such as allocation of hospital resources. Several studies have demonstrated a strong correlation between wastewater SARS-CoV-2 levels and measures of COVID-19 burden, including hospitalizations (5, 6, 8–23). However, most of these studies reported data at the municipal or sub-national level, or over a relatively short period of a few months (5, 6, 8–11, 14, 15, 18, 19, 22, 24–27). Few studies have examined the use of wastewater as a potential correlate of COVID-19 hospitalizations on a national scale. A study in the United States that assessed wastewater surveillance data from 159 counties in 45 states from June 2021 through January 2023 found that wastewater-based models accurately predicted county-level weekly new COVID-19 admissions in the following 1–4 weeks (13). Two additional studies from the United States each found a strong positive correlation between county wastewater virus levels and reported COVID-19 cases from June 2020 to May 2021 (21) and with reported COVID-19 cases and COVID-19 hospitalizations in the upcoming 2 weeks from January to September 2022 (21, 23). A study of Austria’s wastewater surveillance system covering about 70% of the country’s population from April 2020 through August 2022 found that wastewater viral loads were predictive of COVID-19 hospital occupancy, with an average lead time of 9–12 days, allowing prediction of short-term demand for public health services (12).

Here we describe the association between wastewater SARS-CoV-2 levels and COVID-19 hospitalization rate from 2022 through 2024 and explore whether wastewater virus level can predict current and anticipated COVID-19 hospitalization rates at the aggregate national level.

## Methods

### Study design

This study was a retrospective analysis of public health reports of national wastewater surveillance and COVID-19 hospitalizations from 2022 through 2024.

### Inclusion criteria

We conducted a search for national wastewater surveillance and COVID-19 hospitalization data among 27 countries included in a prior study evaluating under-reporting of SARS-CoV-2 infections (28). These 27 countries were selected based on the availability of COVID-19 case report data: Austria, Belgium, Brazil, Canada, Chile, China, Colombia, Denmark, Finland, France, Germany, Greece, Ireland, Israel, Italy, Japan, Luxembourg, Malaysia, the Netherlands, Portugal, Qatar, Singapore, South Africa, Spain, Sweden, the United Kingdom, and the United States.

Our search yielded three countries—Denmark, the Netherlands, and the United States—that met the criteria for sufficient data for analysis, defined as: (1) availability of ≥24 months of national SARS-CoV-2 wastewater surveillance data starting from January 2022 [e.g., United States (24), Denmark (17, 29), and the Netherlands (30)], and (2) availability of data on weekly new COVID-19 hospital admissions and/or hospital occupancy starting from January 2022 (31–33).

### Wastewater SARS-CoV-2 levels

Wastewater SARS-CoV-2 surveillance data were obtained through publicly available country-specific dashboards, reports, and official websites. Countries were included in the wastewater analyses if datasets were downloadable and provided at least 2 years of data starting January 2022. We excluded countries without publicly available national aggregate wastewater values, even if they reported regional or sub-national data. In cases where reported data were not downloadable, the study team contacted publishers of nationally representative wastewater reports.

In Denmark, wastewater SARS-CoV-2 levels are reported as SARS-CoV-2 relative to fecal matter x 10^7^ during January 6, 2022 through September 19, 2024 (29). In the Netherlands, wastewater SARS-CoV-2 levels are reported as average number of SARS-CoV-2 particles x 10^11^ during January 8, 2022 through September 21, 2024 (30). In the United States, wastewater SARS-CoV-2 levels are defined as the number of standard deviations above the baseline wastewater SARS-CoV-2 level, transformed to the linear scale (reported as National Wastewater Viral Activity Level) during January 1, 2022, through September 21, 2024 (34). All wastewater datasets were downloaded on October 28, 2024.

### COVID-19 hospitalizations

Data on COVID-19 hospitalizations were sourced from official public health surveillance reports for all countries with available wastewater surveillance data. Nationally representative measures of hospitalizations were collected, prioritizing data on weekly new hospitalizations expressed as rates per million inhabitants when available. We also supplemented our search of official sources of hospitalization data with data on COVID-19 hospitalization from public repositories including Our World in Data (32).

Hospitalization rates were obtained from the Statens Serum Institut web page for Denmark (29). Netherlands hospitalization rates were obtained from Our World In Data, a publicly available resource that sources hospitalization data from the Netherlands National Coordination Center Patient Distribution (32, 35). In the United States, hospitalization rates were obtained from the Centers for Disease Control and Prevention’s COVID-19 Hospitalization Surveillance Network (COVID-NET) dashboard which monitors laboratory-confirmed, COVID-19-associated hospitalizations (31). All hospitalization datasets were downloaded on October 28, 2024.

### Subvariant predominance

For each country, we defined a subvariant predominance period as any consecutive period when a specific subvariant attained 50% or higher prevalence for ≥4 weeks. Subvariant prevalence was sourced from publicly available databases such as covariants.org or national SARS-CoV-2 surveillance reports. For each country, we defined predominance period by major variant of concern (Omicron) and its major subvariants – BA.1, BA.2, BA.4/5, XBB, or JN.1 (Supplementary Table).

### Data cleaning and exploratory data analyses

Completeness and consistency of variables were evaluated through exploratory analyses. Variables with differing reporting frequencies (e.g., daily vs. weekly) were standardized by converting daily data into trailing weekly sums. For countries without clear day of the week when data are reported (example, “week 1, 2022”), we assigned the weekly value to the Saturday corresponding to the United States CDC “Epi Week” reporting, a widely adopted standard for public health surveillance reporting (36). All COVID-19 hospitalization data were converted to weekly new hospitalization rate per million population using United Nations population estimates in instances where only raw numbers of hospitalizations were available (37). Across the three countries included in this study, there was one missing data point from Denmark (weekly hospitalization rate for the week of April 4, 2024) and two missing data points from the Netherlands (wastewater virus levels for the weeks corresponding to October 8, 2023 and September 29, 2024). Weeks with missing data were excluded from the analysis.

### Scaled wastewater SARS-CoV-2 level

To enable cross-country comparisons, national wastewater SARS-CoV-2 levels were scaled from 0 to 1 by dividing observed levels by the maximum level for each country during the study period. A uniform scale for wastewater SARS-CoV-2 levels partially overcomes the heterogeneity of measurement scales for our data. For example, wastewater SARS-CoV-2 levels were reported as SARS-CoV-2 relative to fecal matter x 10^7^ in Denmark, average number of SARS-CoV-2 particles x 10^11^ in the Netherlands, and number of standard deviations above the baseline wastewater SARS-CoV-2 level, transformed to the linear scale in the United States.

### Statistical analyses

For each country, we calculated descriptive statistics including medians and interquartile ranges for national wastewater virus levels and hospitalization rates for the overall study period and by subvariant predominance. We explored the association between wastewater virus levels and hospitalization rate using line plots, scatterplots, and correlation coefficients. We also assessed different lags between wastewater and hospitalization data for each country overall, and by subvariant predominance, using analysis of peak correlation coefficients (38) within a range of 0–4 weeks lead or lag. For variable combinations with sample sizes ≥8 weeks, we calculated Pearson correlation coefficients along with corresponding 95% confidence intervals (CI) using the *cor* function in R version 4.1 (39). Data consistency and variability were evaluated to identify appropriate statistical transformations, such as logarithmic scaling.

To explore time trends in the association between hospitalization rates and wastewater virus levels, we calculated two measures: (1) *Wastewater to hospitalization ratio* – defined as scaled wastewater SARS-CoV-2 level divided by weekly new COVID-19 hospitalization rate per million, which is a measure of the scaled wastewater virus level for a given weekly new COVID-19 hospitalization rate (2) *Hospitalization multiplier* – defined as the inverse of wastewater to hospitalization ratio. The hospitalization multiplier is a measure of national hospitalization rate for a given scaled wastewater level. For example, a hospitalization multiplier of 100 can be interpreted as a weekly new COVID-19 hospitalization rate that is approximately a hundred times the observed scaled wastewater level. The rationale for a hospitalization multiplier is to generate a simple number that can be applied in real world settings to translate a given scaled wastewater virus level into an expected weekly new COVID-19 hospitalization rate. Medians, interquartile ranges (IQR), and descriptive plots by country and predominant subvariant were generated for these two measures.

We explored whether wastewater SARS-CoV-2 levels were predictive of weekly new COVID-19 hospitalizations by evaluating several country-specific linear regression models using weekly new COVID-19 hospitalization, with or without log-transformation as a response variable, and scaled wastewater SARS-CoV-2 levels, with a lag of 0–4 weeks and log-transformations as the main predictor. We used lags of 0–4 weeks in keeping with previous studies and the available weekly hospitalization rates in all three countries in our study (5, 8, 11, 12). We also explored whether the association between hospitalization and wastewater virus level was consistent over time by including number of days since January 1, 2022 as a covariate in our regression models. We evaluated regression models using Akaike and Bayesian information criteria (AIC and BIC), adjusted R^2^, as well as mean absolute percent error (MAPE) and root mean square error (RMSE). We chose MAPE as the main model selection criterion because it was both scale and model agnostic.

All identified data sources were downloaded on October 28, 2024, and analyzed using R v4.1. Medians and interquartile ranges were calculated using the R *quantile* function and the *cor* function was used for correlation analyses (39).

## Results

### Denmark

**Figure 1:**
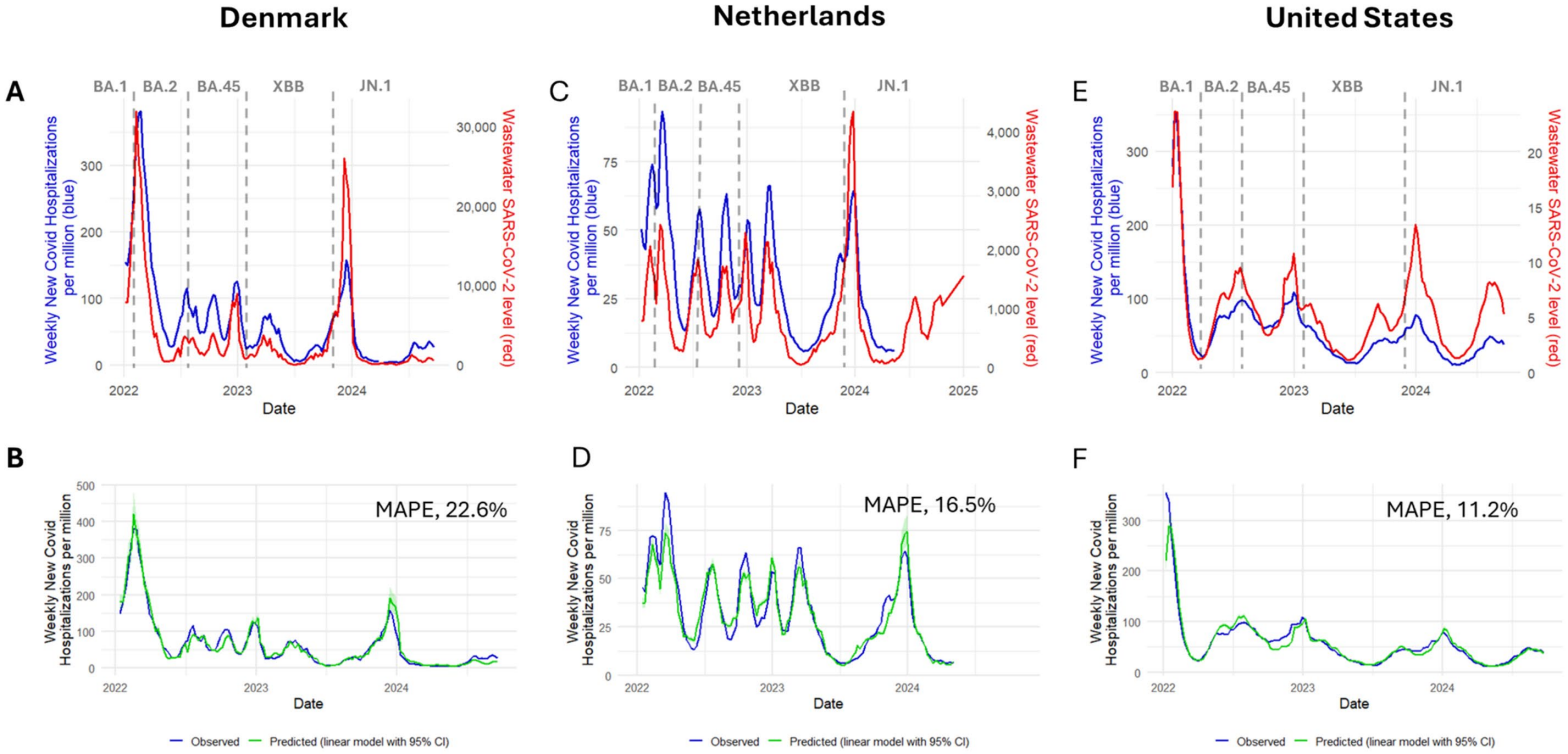
Wastewater SARS-CoV-2 level and weekly new COVID-19 hospitalizations over time **(A,C,E)**, and observed and predicted weekly new COVID-19 hospitalizations **(B,D,F)** in Denmark **(A,B)**, the Netherlands **(C,D)**, and the United States **(E,F)**, 2022–2024. Vertical dashed lines represent start/end dates of Omicron subvariant predominance. Predicted hospitalizations are based on a Model #10 in Tables 5, which is a linear regression model that uses log-transformed, scaled wastewater virus levels with 1 week lag and calendar days as predictors and log-transformed weekly new COVID-19 hospitalizations as outcome. Model coefficients and goodness of fit measures are provided in Table 5 (Model #10). MAPE – mean absolute percentage error for Model #10.

Wastewater SARS-CoV-2 levels (reported as SARS-CoV-2 relative to fecal matter x 10^7^) had a median of 1367.7 × 10^7^ (IQR, 551.3 to 3359.4) during January 6, 2022 through September 19, 2024, with a decreasing trend over time (Table 1; Figure 1A). The highest median wastewater SARS-CoV-2 level was observed during BA.1 predominance (7907.0 × 10^7^; IQR, 7905.4 to 7908.6), followed by BA.2 (7319.2 × 10^7^; IQR, 1210.4 to 17625.7), with the lowest median levels occurring during XBB (1386.4 × 10^7^; IQR, 679.0 to 2396.0) and JN.1 predominance (518.3 × 10^7^; IQR, 196.3 to 925.8). Median weekly new COVID-19 hospitalization rates per million population were 41.0 (IQR, 21.8 to 83.3) for the full study period (Table 1; Figure 1A). The highest COVID-19 hospitalization rates were observed during BA.2 predominance with a median of 163.5 weekly new COVID-19 hospitalizations per million (IQR, 71.9 to 272.0), followed by BA.1 predominance (median 152.1; IQR, 150.9 to 153.2), and lowest during XBB (median 30.2; IQR, 16.6 to 58.1) and JN.1 predominance (median 16.5; IQR, 5.9 to 31.6).

**Table 1:** COVID-19 hospitalization rate and wastewater SARS-CoV-2 level in Denmark, the Netherlands, and the United States, 2022–2024.

Country	Variable	Date range	All Omicron, median (IQR)	By predominant^a^ subvariant, median (IQR)				
				BA.1	BA.2	BA.4/5	XBB	JN.1
Denmark	Weekly new COVID-19 hospitalization rate, per M	January 6, 2022, to September 19, 2024	41.0 (21.8 to 83.3)	152.1 (150.9 to 153.2)	163.5 (71.9 to 272.0)	72.5 (54.8 to 92.6)	30.2 (16.6 to 58.1)	16.5 (5.9 to 31.6)
	Wastewater SARS-CoV-2 level^b^	January 6, 2022, to September 19, 2024	1367.7 (551.3 to 3359.4)	7907.0 (7905.4 to 7908.6)	7319.2 (1210.4 to 17625.7)	2386.7 (1516.3 to 3357.3)	1386.4 (679.0 to 2396.0)	518.3 (196.3 to 925.8)
The Netherlands	Weekly new COVID-19 hospitalization rate, per M	January 8, 2022, to May 4, 2024	28.8 (14.5 to 46.9)	57.7 (48.8 to 70.0)	47.9 (19.9 to 71.0)	32.2 (24.9 to 52.8)	23.3 (11.1 to 38.7)	12.9 (7.3 to 33.5)
	Wastewater SARS-Cov-2 level^c^	January 8, 2022, to September 21, 2024	706.0 (343.0 to 1228.5)	1422.0 (1014.8 to 1663.5)	591.0 (330.0 to 1629.2)	1030.0 (662.0 to 1565.0)	574.0 (266.0 to 1130.0)	586.0 (147.5 to 1072.0)
United States	Weekly new COVID-19 hospitalization rate, per M	January 1, 2022, to September 21, 2024	45.0 (29.0 to 71.0)	149.0 (66.5 to 277.5)	29.5 (25.0 to 41.0)	77.5 (70.8 to 94.0)	41.0 (22.8 to 48.2)	35.0 (18.0 to 45.0)
	Wastewater SARS-CoV-2 level^d^	January 1, 2022, to September 21, 2024	5.0 (2.7 to 7.2)	9.2 (3.2 to 18.1)	1.8 (1.3 to 3.3)	6.7 (5.0 to 8.3)	4.3 (2.1 to 5.6)	5.4 (2.3 to 7.8)

CDC, Centers for Disease Control and Prevention; IQR, interquartile range; M, million. ^a^Subvariant predominance period defined as any consecutive period when a specific subvariant exceeded 50% prevalence for ≥4 weeks as presented in Table 5. ^b^SARS-CoV-2 relative to fecal matter x 107. ^c^Average number of SARS-CoV-2 particles x 10^11^. ^d^National Wastewater Viral Activity Level as defined by the CDC.

### The Netherlands

In the Netherlands, wastewater SARS-CoV-2 level (reported as average number of SARS-CoV-2 particles x 10^11^) had a median of 706.0 (IQR, 343.0 to 1228.5) during January 8, 2022 through September 21, 2024 (Table 1; Figure 1C) (30, 40). Wastewater virus levels were highest during BA.1 predominance (1422.0 × 10^11^; IQR, 1014.8 to 1663.5), followed by BA.4/5 (1030.0 × 10^11^; IQR, 662.0 to 1565.0), and were lowest during XBB (574.0 × 10^11^; IQR, 266.0 to 1130.0), and JN.1 predominance (586.0 × 10^11^; IQR, 147.5 to 1072.0). Median weekly new COVID-19 hospitalization rate per million was 28.8 (IQR, 14.5 to 46.9) during January 8, 2022, through May 4, 2024 (Table 1; Figure 1C). Hospitalization rates peaked during BA.1 predominance with 57.7 new COVID-19 hospitalizations per million (IQR, 48.8 to 70.0), followed by 47.9 per million (IQR, 19.9 to 71.0) during BA.2 predominance, and were lowest during XBB (23.3 per million; IQR, 11.1 to 38.7) and JN.1 predominance (12.9 per million; IQR, 7.30 to 33.5).

### United States

In the United States, wastewater SARS-CoV-2 level is defined as the number of standard deviations above the baseline wastewater SARS-CoV-2 level, transformed to the linear scale (reported as National Wastewater Viral Activity Level) (24). Median wastewater SARS-CoV-2 level was 5.0 (IQR, 2.7 to 7.2) during January 1, 2022, through September 21, 2024 (Table 1; Figure 1E). The highest levels were observed during BA.1 predominance (median, 9.2; IQR, 3.2 to 18.1), followed by BA.4/5 (median, 6.7; IQR, 5.0 to 8.3), JN.1 (median, 5.4; IQR, 2.3 to 7.8), and XBB (median, 4.3; IQR, 2.1 to 5.6). Median weekly new COVID-19 hospitalization rate per million population was 45.0 (IQR, 29.0 to 71.0) during January 1, 2022 through September 21, 2024 (Table 1; Figure 1E) (31). Hospitalization rates peaked during BA.1 predominance (149.0 per million; IQR, 66.5 to 277.5), followed by BA.4/5 (77.5 per million; IQR, 70.8 to 94.0), and were lowest during JN.1 (35.0 per million; IQR, 18.0 to 45.0) and BA.2 predominance 29.5 (25.0 to 41.0).

### Hospitalization multipliers

**Figure 2:**
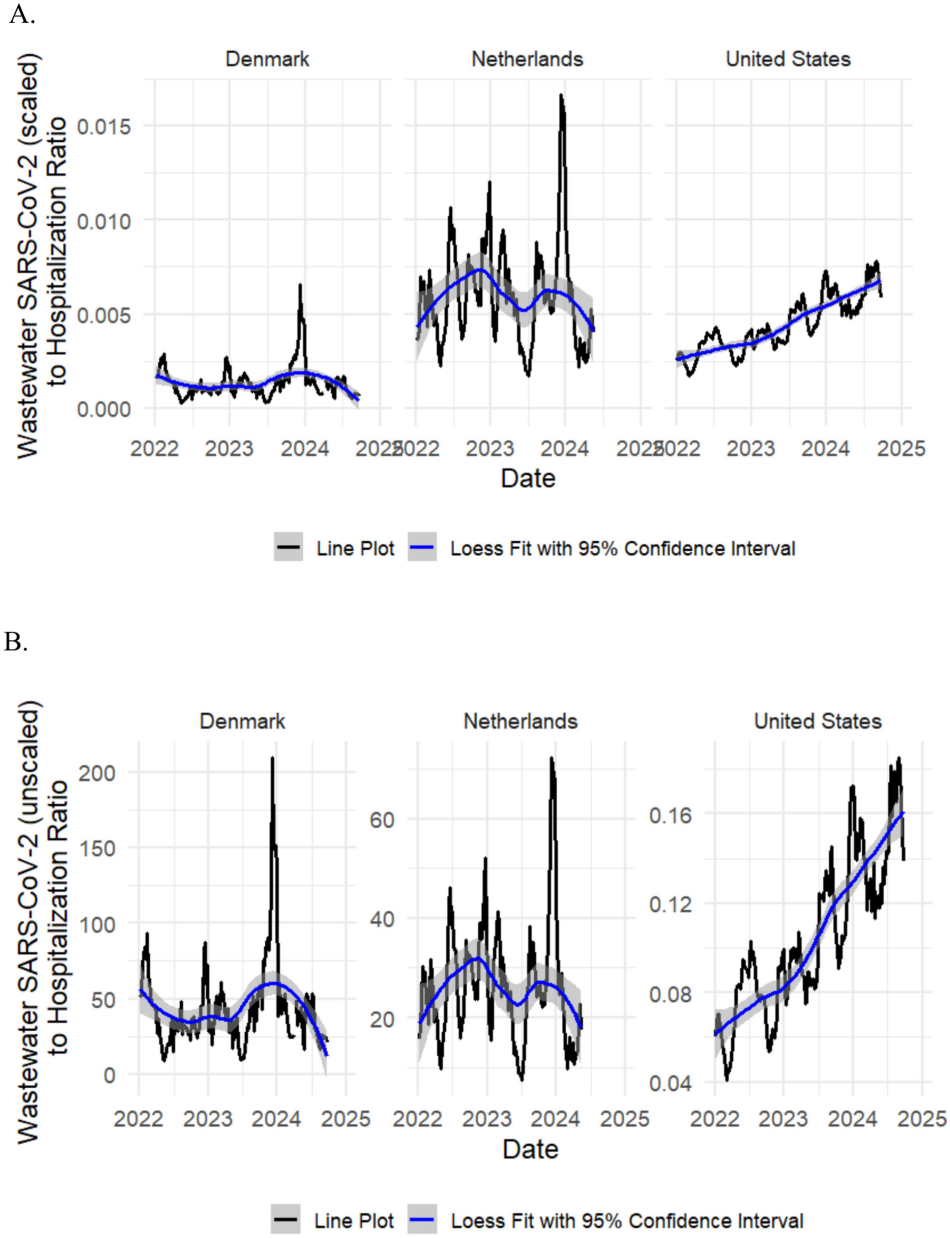
Ratio of scaled **(A)** and unscaled **(B)** wastewater SARS-CoV-2 level to weekly new COVID-19 hospitalization rate per million in Denmark, the Netherlands, and the United States, 2022–2024.

**Figure 3:**
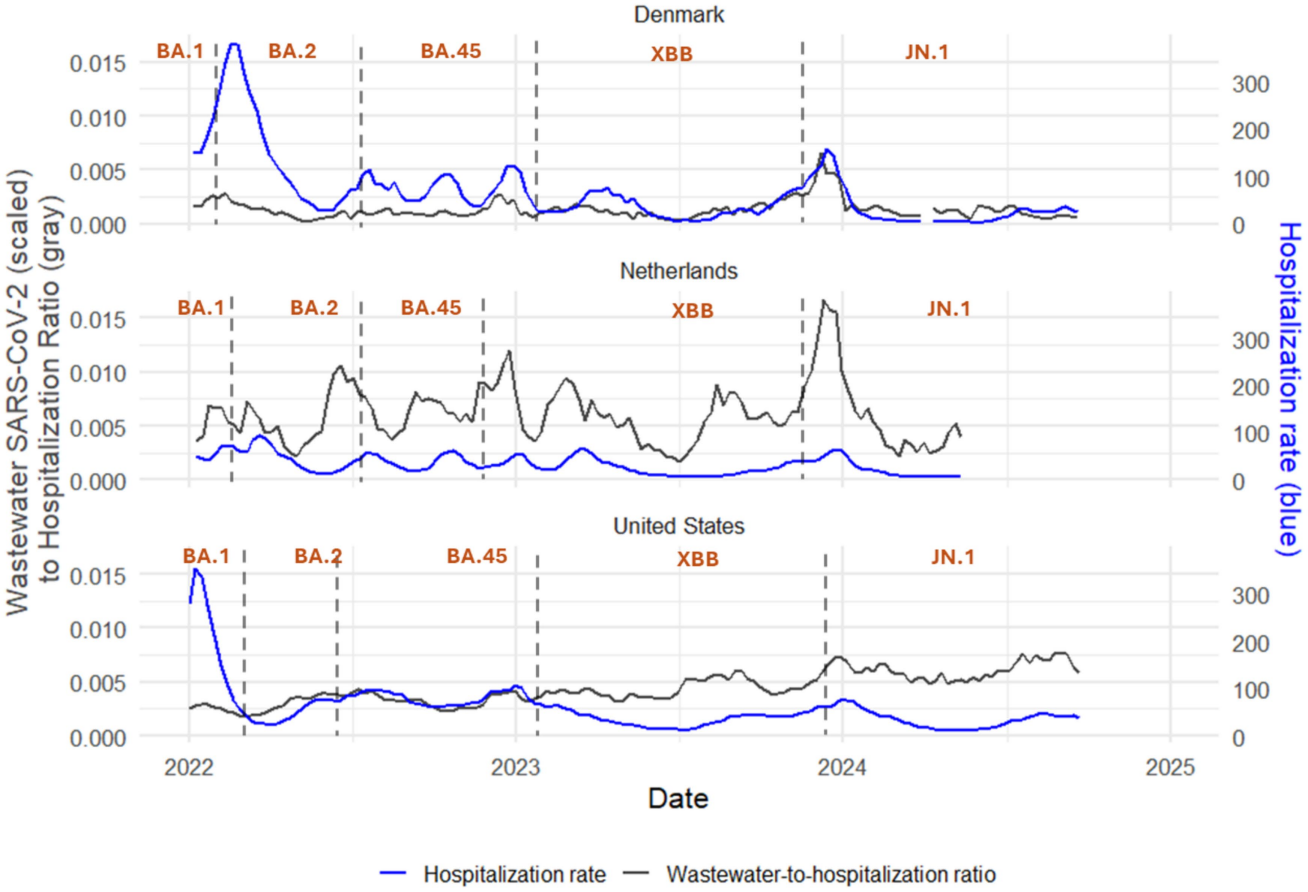
Line charts of ratio of scaled wastewater SARS-CoV-2 level to weekly new COVID-19 hospitalization rate per million (gray, left y-axis) with weekly new COVID-19 hospitalization rate per million (blue, right y-axis) in Denmark, the Netherlands, and the United States, 2022–2024. Vertical dashed lines represent start/end dates of Omicron subvariant predominance.

The median hospitalization multipliers using scaled wastewater SARS-CoV-2 level were 859.3 (IQR, 621.7 to 1210.4), 178.3 (IQR, 133.7 to 243.7), and 245.9 (IQR, 184.0 to 293.7) for Denmark, the Netherlands, and the United States, respectively, during the study period (Table 3; for multipliers using unscaled wastewater SARS-CoV-2 level, see Table 4). There was no clear temporal trend in median hospitalization multiplier in Denmark and the Netherlands with a potential trend of decreasing median multipliers over time in the United States (Table 3). In Denmark, median hospitalization multipliers peaked during BA.4/5 predominance (998.5; IQR, 831.2 to 1118.6) and were lowest during BA.1 predominance (613.0; IQR, 608.6 to 617.3). In the Netherlands, median hospitalization multipliers peaked during BA.2 predominance (226.6; IQR, 81.8 to 298.0) and were lowest during BA.4/5 predominance (138.4; IQR, 121.1 to 183.6). In the United States, median hospitalization multipliers peaked at 393.3 (IQR, 360.9 to 485.5) during BA.1 predominance and were lowest during JN.1 predominance at 167.1 (IQR, 144.5 to 184.6). Loess plots (Figures 2, 3) also show no clear temporal trend in wastewater to hospitalization ratio in Denmark and the Netherlands with potential trend of increasing wastewater to hospitalization ratios in the United States.

### Correlation analysis

There was a strong correlation (Pearson correlation coefficients ≥0.80) between weekly new COVID-19 hospitalization rate and wastewater SARS-CoV-2 level in all three countries (Table 2). In Denmark, the correlation coefficient was 0.86 (95% CI, 0.82 to 0.90), in the Netherlands it was 0.80 (95% CI, 0.72 to 0.85), and in the United States it was 0.89 (95% CI, 0.85 to 0.92). Correlations were consistently strong across all subvariant predominance periods in all three countries (range, 0.72 to 1.0). Across all Omicron subvariant predominance periods, correlations in the United States remained consistently high (≥0.91), while correlations in Denmark showed more variability, with the strongest correlation during BA.2 (0.94, 95% CI: 0.85 to 0.98) and JN.1 (0.94, 95% CI: 0.89 to 0.97) but a relatively weaker correlation during BA.4/5 (0.72, 95% CI: 0.49 to 0.85). In the Netherlands, correlations were lowest during BA.1 (0.76, 95% CI: 0.12 to 0.95) and highest during JN.1 predominance (0.96, 95% CI: 0.90 to 0.98).

**Table 2:** Correlation, peak correlation and lead/lag between wastewater SARS-CoV-2 level and weekly new COVID-19 hospitalization rate per million in Denmark, the Netherlands, and the United States, overall and by predominant subvariant, 2022–2024.

Country	Measure	Correlation coefficient (95% CI)/# of weeks lead or lag^a^					
		All Omicron	BA.1	BA.2	BA.4/5	XBB	JN.1
Denmark	Correlation^b^	0.86 (0.82 to 0.90)	NA	0.94 (0.85 to 0.98)	0.72 (0.49 to 0.85)	0.85 (0.73 to 0.92)	0.94 (0.89 to 0.97)
	Peak correlation^c^	0.88 (0.84 to 0.91)	NA	0.98 (0.95 to 0.99)	0.78 (0.60 to 0.89)	0.89 (0.80 to 0.94)	0.94 (0.89 to 0.97)
	Lead or lag^d^	Lag 1 week	NA	Lag 1 week	Lag 1 week	Lag 2 weeks	No lead or lag
The Netherlands	Correlation	0.80 (0.72 to 0.85)	0.76 (0.12 to 0.95)	0.91 (0.75 to 0.97)	0.81 (0.61 to 0.91)	0.90 (0.83 to 0.94)	0.96 (0.90 to 0.98)
	Peak correlation	0.83 (0.76 to 0.88)	0.76 (0.12 to 0.95)	0.98 (0.93 to 0.99)	0.88 (0.75 to 0.95)	0.96 (0.92 to 0.98)	0.96 (0.90 to 0.98)
	Lead or lag	Lag 1 week	No lead or lag	Lag 1 week	Lag 1 week	Lag 1 week	Lag 1 week
United States	Correlation	0.89 (0.85 to 0.92)	1.00 (0.98 to 1.00)	0.99 (0.93 to 1.00)	0.93 (0.87 to 0.96)	0.91 (0.84 to 0.95)	0.98 (0.97 to 0.99)
	Peak correlation	0.89 (0.85 to 0.92)	1.00 (0.98 to 1.00)	0.99 (0.93 to 1.00)	0.93 (0.87 to 0.96)	0.94 (0.90 to 0.97)	0.98 (0.97 to 0.99)
	Lead or lag	No lead or lag	No lead or lag	No lead or lag	No lead or lag	Lag 2 weeks	No lead or lag

CI, confidence interval. NA, not applicable. ^a^Any subvariant with <8 weeks of available data is excluded from this analysis. ^b^Pearson correlation coefficient between wastewater SARS-CoV-2 level and hospitalization rate without lead or lag. ^c^Maximum observed correlation coefficient between wastewater SARS-CoV-2 level and hospitalization rate regardless of lead or lag. ^d^Lead or lag (in weeks) between wastewater SARS-CoV-2 and hospitalization that corresponded with peak correlation.

**Table 3:** Multipliers for weekly new COVID-19 hospitalization rate per million using scaled wastewater SARS-CoV-2 level by country and predominant variant.

Country	Median (interquartile range)					
	All Omicron	BA.1	BA.2	BA.4/5	JN.1	XBB
Denmark	859.3 (621.7 to 1210.4)	613.0 (608.6 to 617.3)	757.3 (509.1 to 1623.9)	998.5 (831.2 to 1118.6)	825.3 (629.2 to 1288.1)	831.6 (614.5 to 1132.5)
The Netherlands	178.3 (133.7 to 243.7)	191.8 (150.7 to 234.3)	226.6 (181.8 to 298.0)	138.4 (121.1 to 183.6)	224.5 (137 to 312.4)	173.1 (134.2 to 238.6)
United States	245.9 (184.0 to 293.7)	393.3 (360.9 to 485.5)	369.4 (291.4 to 430.6)	292.6 (258.0 to 338.2)	167.1 (144.5 to 184.6)	238.7 (193.2 to 267.1)

**Table 4:** Multipliers for weekly new COVID-19 hospitalization rate per million using wastewater SARS-CoV-2 level (unscaled) by country and predominant variant.

Country	Median (interquartile range)					
	All Omicron	BA.1	BA.2	BA.4/5	XBB	JN.1
Denmark	0.027 (0.0195 to 0.038)	0.0192 (0.0191 to 0.0194)	0.0238 (0.016 to 0.0509)	0.0313 (0.0261 to 0.0351)	0.0261 (0.0193 to 0.0355)	0.0259 (0.0197 to 0.0404)
The Netherlands	0.0411 (0.0308 to 0.0561)	0.0442 (0.0347 to 0.054)	0.0522 (0.0419 to 0.0686)	0.0319 (0.0279 to 0.0423)	0.0399 (0.0309 to 0.0549)	0.0517 (0.0315 to 0.0719)
United States	10.3704 (7.7622 to 12.3878)	16.5874 (15.2219 to 20.4751)	15.5808 (12.2908 to 18.1603)	12.3392 (10.883 to 14.2619)	10.0655 (8.1476 to 11.2632)	7.0483 (6.0947 to 7.785)

**Table 5:** Linear regression models for weekly new COVID hospitalization rate per million in Denmark, the Netherlands and the United States, 2022–2024.

Country	Response	Predictors	Regression model coefficients				Model accuracy and goodness of fit					
			Model ID	Intercept	Beta1	Beta2	MAPE	RMSE	AIC	BIC	Adj. R^2^	CV
Denmark	Hospitalization rate	Wastewater	1	26.3117	335.8185		101.5320	36.3183	1409.1532	1417.9781	0.7462	1469.2126
		Wastewater (1-week lag)	2	25.0774	341.6778		95.8298	34.2718	1392.9130	1401.7380	0.7740	1313.0483
		Log(wastewater)	3	191.3050	40.3033		135.5194	43.6578	1460.6898	1469.5147	0.6332	2015.7790
		Log[wastewater (1-week lag)]	4	190.8308	40.4189		141.2391	43.2823	1458.2710	1467.0959	0.6395	1984.1086
		Calendar days	5	140.5708	−0.1517		120.6669	58.0436	1540.4370	1549.2619	0.3516	3506.5445
	Log(hospitalization rate)	Wastewater	6	3.1604	4.0236		96.0077	107.0464	352.4933	361.3182	0.4429	0.7216
		Wastewater (1-week lag)	7	3.1483	4.0706		95.7711	108.2213	349.6193	358.4442	0.4542	0.7069
		Log(wastewater)	8	5.9145	0.7301		35.9742	35.0474	151.4454	160.2704	0.8675	0.1709
		Log[wastewater (1-week lag)]	9	5.9101	0.7336		32.4986	32.9625	138.3727	147.1976	0.8793	0.1558
		Log[wastewater (1-week lag)]; calendar days	10	6.0938	0.6309	−0.0010	22.5803	14.7158	68.5334	80.3000	0.9272	0.0948
		Log[wastewater (2-week lag)]	11	5.8040	0.7039		41.6413	38.2575	195.4880	204.2914	0.8160	0.2364
		Log[wastewater (2-week lag)]; calendar days	12	6.0079	0.5947	−0.0011	33.4587	22.5483	148.0983	159.8362	0.8701	0.1684
		Log[wastewater (4-week lag)]	13	5.3967	0.5824		73.6441	53.7921	304.5415	313.3014	0.5757	0.5348
		Log[wastewater (4-week lag)]; calendar days	14	5.6799	0.4423	−0.0014	62.3349	42.6146	272.9827	284.6626	0.6654	0.4250
The Netherlands	hospitalization rate	Wastewater	1	13.1675	88.6971		43.1179	12.6943	948.5023	956.8397	0.6370	176.8091
		Wastewater (1-week lag)	2	12.3352	91.9854		38.4411	11.9178	933.4799	941.8173	0.6800	158.2523
		Log(wastewater)	3	65.2404	16.5128		42.3429	11.4934	924.8483	933.1857	0.7024	136.6031
		Log[wastewater (1-week lag)]	4	66.0796	17.0768		41.5510	10.8213	910.5092	918.8465	0.7362	121.5412
		Calendar days	5	52.6212	−0.0464		73.8507	17.7938	1028.8739	1037.2113	0.2867	327.9935
	Log(hospitalization rate)	Wastewater	6	2.5426	3.1236		45.0816	32.6910	173.9112	182.2485	0.5934	0.2621
		Wastewater (1-week lag)	7	2.5189	3.2137		43.7820	34.5564	164.7967	173.1341	0.6234	0.2452
		Log(wastewater)	8	4.5418	0.6646		24.4139	11.3195	49.2661	57.6035	0.8573	0.0869
		Log[wastewater (1-week lag)]	9	4.5790	0.6890		19.5775	10.4619	3.2713	11.6086	0.9031	0.0593
		Log[wastewater (1-week lag)]; calendar days	10	4.7158	0.6287	−0.0006	16.4553	7.0263	−36.1272	−25.0107	0.9310	0.0425
		Log[wastewater (2-week lag)]	11	4.5395	0.6757		22.3577	13.0112	48.5837	56.8957	0.8583	0.0871
		Log[wastewater (2-week lag)]; calendar days	12	4.7004	0.6100	−0.0007	19.4164	9.8779	16.3881	27.4709	0.8930	0.0667
		Log[wastewater (4-week lag)]	13	4.3142	0.5760		42.9242	18.5462	168.8394	177.1002	0.5988	0.2461
		Log[Wastewater (4-week lag)]; calendar days	14	4.5717	0.4869	−0.0010	38.2390	15.8497	146.6682	157.6825	0.6714	0.2034
United States	Hospitalization rate	Wastewater	1	−6.9465	277.0964		31.0861	23.3001	1303.1409	1312.0084	0.7762	602.5052
		Wastewater (1-week lag)	2	−4.8622	264.1106		28.7247	24.5944	1318.4938	1327.3612	0.7507	666.2846
		Log(wastewater)	3	143.9635	51.7814		47.5568	34.9501	1418.2925	1427.1600	0.4965	1314.0691
		Log[wastewater (1-week lag)]	4	142.7768	51.3250		46.6604	34.7918	1417.0028	1425.8703	0.5010	1294.4332
		Calendar days	5	100.9702	−0.0880		64.1076	42.4949	1473.8038	1482.6713	0.2556	1893.3982
	Log(hospitalization rate)	Wastewater	6	2.9698	3.5888		35.0877	47.6151	142.3462	151.2137	0.6734	0.1600
		Wastewater (1-week lag)	7	2.9800	3.4926		34.8754	46.6866	139.7994	148.6668	0.6793	0.1575
		Log(wastewater)	8	5.2903	0.8882		28.5787	27.3244	98.4463	107.3138	0.7603	0.1160
		Log[wastewater (1-week lag)]	9	5.2771	0.8846		27.4228	27.7922	89.6188	98.4863	0.7747	0.1089
		Log[wastewater (1-week lag)]; calendar days	10	5.6803	0.8205	−0.0010	11.2378	14.8143	−130.3849	−118.5616	0.9525	0.0230
		Log[wastewater (2-week lag)]	11	5.1748	0.8309		28.1959	25.6548	106.9636	115.8099	0.7284	0.1235
		Log[wastewater (2-week lag)]; calendar days	12	5.5514	0.7676	−0.0010	17.5296	17.9373	−21.3438	−9.5487	0.8914	0.0497
		Log[wastewater (4-week lag)]	13	4.8518	0.6519		38.3401	22.8450	173.0608	181.8642	0.5041	0.2005
		Log[wastewater (4-week lag)]; calendar days	14	5.1863	0.5871	−0.0009	32.5134	20.2407	126.6521	138.3900	0.6474	0.1440

Hospitalization rate – weekly new COVID-19 hospitalization rate per million; wastewater – wastewater SARS-CoV-2 level, scaled; calendar days – days since January 1, 2022. AIC, Akaike information criterion; Adj. R^2^, adjusted R^2^; BIC, Bayesian information criterion; CV, leave-one-out cross-validation statistic; MAPE, mean absolute percent error; NA – not available; RMSE, root mean squared error.

The peak correlation between weekly hospitalization rate and wastewater for Denmark and the Netherlands occurred at a lag of 1 week while peak correlation for the United States had no lead or lag (Table 2). However, linear regression models suggested a 1-week lag between hospitalization rate and wastewater virus levels in all three countries (Table 5).

### Regression models

**Figure 4:**
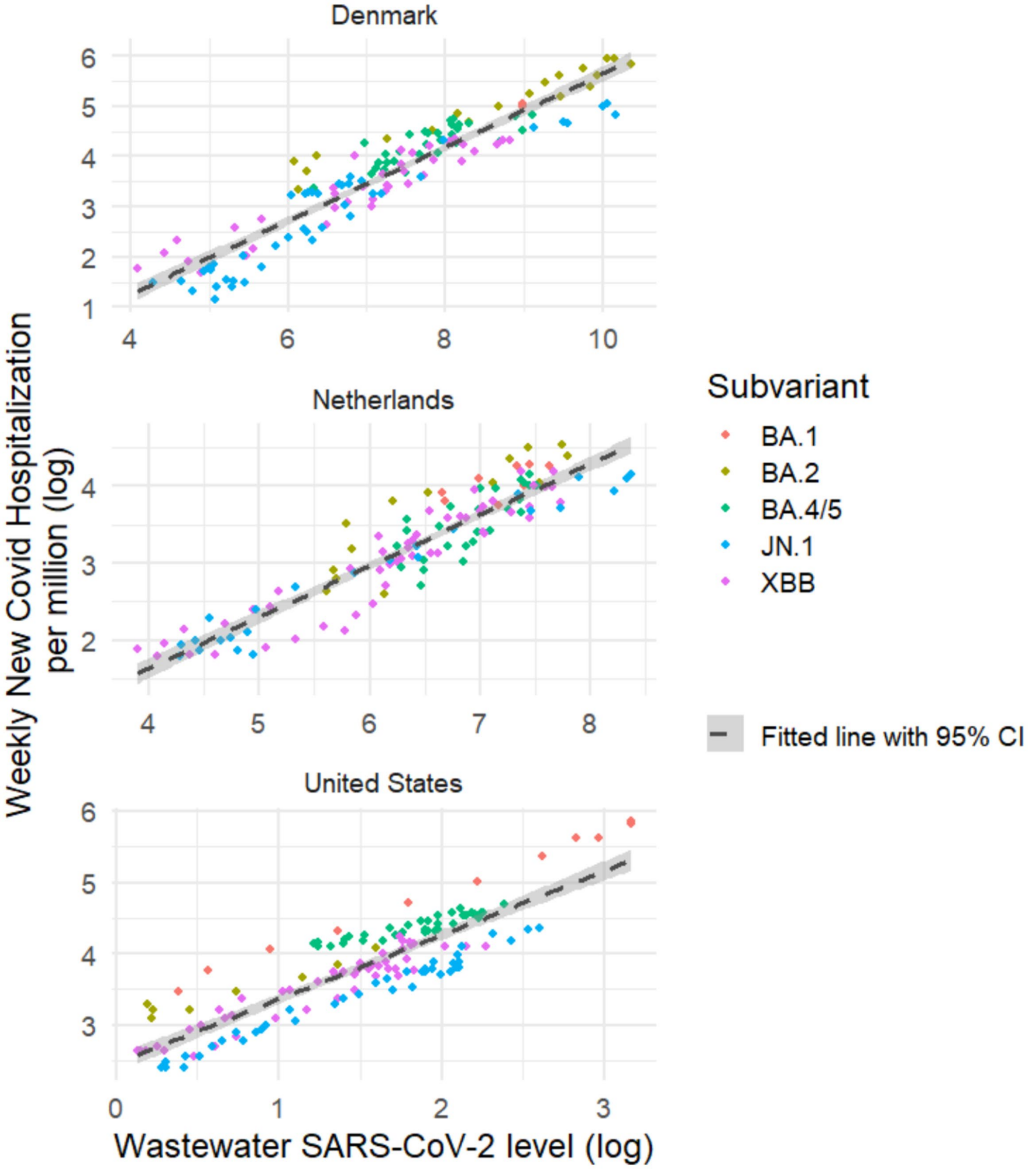
Weekly new COVID-19 hospitalizations (log-transformed) and wastewater SARS-CoV-2 level (log-transformed) by predominant subvariant.

For Denmark, the Netherlands, and the United States, the best fitting regression model used log-transformed weekly new COVID-19 hospitalization rate as response variable with log-transformed wastewater virus level from the previous week and calendar days as covariates (Table 5; Figures 1B,D,F, 4). Model intercepts (6.1, 4.7, and 5.7, for Denmark, the Netherlands and the United States, respectively), regression coefficients for wastewater virus level (0.63, 0.63, and 0.82, for Denmark, the Netherlands and the United States, respectively), and calendar days (−0.0010, −0.0006 and −0.0010 for Denmark, the Netherlands and the United States, respectively) were generally similar across the three countries in our study. These models suggest that (1) log-transformed weekly hospitalization rate increases as the log-transformed previous-week wastewater SARS-CoV-2 level increases, and (2) in all three countries, there was a statistically significant decrease in log-transformed weekly hospitalization rate over time after adjusting for wastewater virus level.

The best fitting regression model was the same for all three countries (model 10 in Table 5, Supplementary Figures 1–3) with high adjusted R^2^ values (>0.9) and MAPE of 22.6, 16.5 and 11.2% for Denmark, the Netherlands, and the United States, respectively. Increasing the wastewater lag to 2–4 weeks reduced model accuracy but still provided moderately accurate predictions. For example, increasing the wastewater lag to 2 weeks (4 weeks) increased MAPE to 33.5, 19.4, and 17.5% (62.3, 38.2, and 32.5%), in Denmark, the Netherlands and the United States, respectively (models 12 and 14 in Table 5).

## Discussion

In this study, we evaluated the association between levels of SARS-CoV-2 in wastewater and COVID-19 hospitalization rates at the national level from January 2022 through September 2024 (encompassing both pandemic and post-pandemic periods) in Denmark, the Netherlands, and the United States. We found a strong positive correlation between wastewater SARS-CoV-2 levels and weekly new COVID-19 hospitalization rates in all three countries with overall correlation coefficients ranging from 0.80 to 0.89. Correlation coefficients ranged from 0.72 to 1.0 during BA.1, BA.2, BA.4/5, XBB, and JN.1 predominance periods. Correlation coefficients were consistently high in the United States (>0.9) during all subvariant predominance periods while there was some variation in correlation coefficients in Denmark (range 0.72 to 0.94) and the Netherlands (range, 0.76 to 0.96). These differences may reflect variation in national testing intensity, hospitalization admission criteria, or the response of health systems during different phases of the pandemic. Taken together, these results suggest that SARS-CoV-2 wastewater virus levels are a reliable predictor of COVID-19 hospitalization rates both during and after the pandemic and regardless of changes in circulating variants, even though the degree of correlation of wastewater to hospitalization dynamically changes during the different waves of infection. These findings align with several studies reporting strong positive correlations between wastewater virus levels and COVID-19 hospitalizations across diverse geographic settings and variant predominance (5, 8, 11–15, 41).

We also observed that the peak correlation between wastewater SARS-CoV-2 levels and weekly hospitalizations occurred with a one-week lag in Denmark and Netherlands while peak correlation coefficients occurred with no lead or lag in the United States. In all three countries, the best fitting linear regression models used a 1-week lag between hospitalization and wastewater virus levels, and included log-transformed, scaled, wastewater virus level and calendar days as predictors and log-transformed hospitalization rate as outcome. This model performed best overall across Omicron sub-variant predominance periods and across geographical regions. Taken together, our findings suggest a lag between wastewater virus level and hospitalization rate of 1 week for Denmark and Netherlands and 0–1 week for the United States. Our results are consistent with previous studies of wastewater SARS-CoV-2 levels and hospitalization, which reported lags of 4 to 12 days (5, 8, 11, 12, 14, 15, 42). The reason for a lag between hospitalization and wastewater SARS-CoV-2 levels likely reflects the natural history of SARS-CoV-2 infection, with virus shedding beginning at the presymptomatic and early symptomatic stage, and severe symptoms that would require hospitalization generally occurring days after initial symptoms begin (43). Another potential reason for a lag between wastewater and hospitalization rates could be reporting delays in hospitalization data.

Hospitalization multipliers (the hospitalization rate for a given scaled wastewater virus level) were highly variable across countries with medians of 859.3 (IQR, 621.7 to 1210.4), 178.3 (IQR, 133.7 to 243.7), and 245.9 (IQR, 184.0 to 293.7) in Denmark, the Netherlands and the United States, respectively. Regression models, however, showed a statistically significant decrease in hospitalization rate relative to wastewater virus levels over time in all three countries during the study period. The trend of decreasing hospitalization rates relative to wastewater virus levels is likely due to an increase in population immunity from immunizations and infection over time (1). Alternatively, the decreasing hospitalization could also be due to improvements in outpatient treatment of infected cases and/or decreasing virulence of recent SARS-CoV-2 variants (2).

This finding is consistent with a study from Florida (USA) that found total COVID-19 hospitalizations relative to wastewater were highest during the initial infection wave in 2020 followed by Delta and Omicron waves (10). Although a subsequent study conducted in California reported that wastewater to hospitalization ratios were relatively consistent across two Omicron waves of infections (a likely Omicron BA.1/BA.2 wave from December 2021 through March 2022 and a likely BA.4/5 wave from April through September 2022) (8). Discrepancies between these studies could be a result of their limited geographic scope or differences in time periods. Namely, while rates of hospitalization have fallen over time, there may not have been dramatic differences in rates of hospitalization between the first two Omicron waves. Regardless, our finding of decreased hospitalizations relative to wastewater virus levels over time in Denmark, the Netherlands, and the United States is likely a result of increasing population immunity to SARS-CoV-2, improved early treatment of COVID-19, or both (44). Hospitalization multipliers are easy to compute and interpret and could serve as a contemporaneous measure of disease burden. A knowledge of historical multipliers for any given country can be applied to the wastewater level today to calculate expected hospitalization burden. In essence, hospitalization multipliers enable public health professionals to rapidly translate wastewater surveillance signals into expected healthcare demand, thereby supporting proactive resource allocation. In summary, wastewater surveillance data offers a population-level signal of infection trends that precedes increases in hospitalizations, and is independent of clinical testing rate, thereby supporting timely public health decision-making and resource planning.

### Limitations

This study has several limitations. First, it focused on three countries with publicly available national wastewater surveillance data, limiting the generalizability of the findings. Additional studies from a broader range of countries, particularly low- and middle-income settings, are needed to understand the global association between wastewater surveillance and COVID-19 hospitalizations (45). While scaling SARS-CoV-2 wastewater concentrations to each country’s observed maximum enables within-country temporal comparisons, differences in assay type, viral target regions, sample processing protocols, and normalization strategies limit the interpretation of results across settings. Likewise, differences in hospitalization rates may be due to heterogeneity in hospitalization definitions, availability of testing, population immunity levels, and vaccination coverage across countries during the study period. Additional factors that may explain differences in hospitalization multipliers include differences in population composition such as age, and immune status, differences in vaccine coverage, prior infections and prevailing SARS-CoV-2 variants (38, 46–49). For example, the U. S. reports SARS-CoV-2 wastewater virus levels as deviations from historical baselines, whereas Denmark and the Netherlands use absolute viral load measures relative to fecal content. These discrepancies may partially account for the observed differences in hospitalization multipliers and model coefficients. For example, both regression and correlation analysis found a 1-week lag between wastewater virus levels and hospitalization rate in Denmark and Netherlands while in the United States, regression analysis found a lag of 1 week in contrast to correlation analysis that found no lead or lag. While it is reasonable to assume that the true lag in the United States is somewhere between 0 and 7 days, it is difficult to determine whether the true lag is closer to 7 days.

Second, the aggregation of data at the national level on a weekly time scale may mask regional variations or localized spikes in infections and hospitalizations. In addition, efforts to make wastewater data comparable across jurisdictions do not capture underlying differences in sample collection, laboratory methods, and data aggregation. Developing standardized protocols for wastewater data collection, analysis, and reporting is essential for improving comparability. Similarly, there are subtle differences in how COVID-19 hospitalizations are defined, with the US reporting weekly new COVID-19 hospitalization rate based on a representative sample of the US population, while hospitalization rates in Denmark and the Netherlands were based on total number of weekly new COVID-19 hospitalizations at the national level which may include both patients with COVID-19 as their primary cause of hospitalization and patients with incidental infections (2, 40). In Netherlands, it is reported that only about 54 to 78% of hospitalized SARS-CoV-2-infected patients had COVID-19 as the primary or a secondary reason for admission (40). Another potential source of uncertainty is within country changes in number of wastewater monitoring sites and refinements of laboratory and/or statistical methods. For example, the proportion of the U. S. population served by national wastewater surveillance sites increased from 12% in 2020 to 45% in 2022 (24). In Denmark, the number of sampling sites declined from 202 in April 2022 to 29 in April 2024 with corresponding reduction in population coverage from 85 to 48% (50).

Third, while hospitalization multipliers can provide important information on real-time COVID-19 burden in real world settings, these multipliers are context specific and not generalizable between different settings. Also, hospitalization multipliers do not account for temporal changes in the relationship between wastewater virus level and hospitalization driven by changes in surveillance systems and/or changes in disease severity over time. Thus, hospitalization multipliers should be ideally supplemented with additional epidemiologic data including test positivity rate, emergency department and/or urgent care visits and context-specific modeling.

Fourth, we used linear regression models with a limited number of variables, namely, scaled wastewater virus level and calendar days to predict national COVID-19 hospitalization rate. Our model could potentially be improved with additional epidemiological variables and/or more complex modelling approaches.

### Conclusion

SARS-CoV-2 wastewater virus levels were strongly correlated with COVID-19 hospitalization rates in the following week, providing a cost-effective method for real-time COVID-19 tracking in the post-pandemic era as global and national COVID-19 surveillance systems continue to wind down.

## Data Availability

Publicly available datasets were analyzed in this study. This data can be found at: United States CDC Wastewater Monitoring https://www.cdc.gov/nwss/ US CDC Hospitalization https://www.cdc.gov/covid/php/covid-net/index.html Denmark National Surveillance https://en.ssi.dk/surveillance-and-preparedness/surveillance-in-denmark/covid-19/national-surveillance-of-sars-cov-2-in-wastewater Netherlands wastewater surveillance https://www.rivm.nl/en/coronavirus-covid-19/research/wastewater#sewageresearch Our World in Data https://covid-19.nyc3.digitaloceanspaces.com/public/owid-covid-data.csv.
