# Supplementary material for: Association between SARS-CoV-2 in wastewater and COVID-19 hospitalizations in three countries, 2022–2024
Source: Front Public Health. 2025 Dec 18;13:1679596. doi: 10.3389/fpubh.2025.1679596 (PMC12756388; doi:10.3389/fpubh.2025.1679596)
Supplement: Supplementary file 1 [file Data_Sheet_1.pdf]

SUPPLEMENTARY MATERIALS

Supplementary Table: Subvariant predominance periods by country.

**Country**	**BA.1**	**BA.2**	**BA.4/5**	**XBB**	**JN.1**
Denmark	2021-12-20 to 2022-01-16	2022-01-17 to 2022-06-05	2022-06-06 to 2023-01-15	2023-02-27 to 2023-11-19	2023-11-20 to 2024-09-21
The Netherlands	2022-01-03 to 2022-02-27	2022-02-28 to 2022-06-05	2022-06-06 to 2022-12-04	2022-12-05 to 2023-11-19	2023-11-20 to 2024-09-21
United States	2021-12-20 to 2022-03-13	2022-03-14 to 2022-05-08	2022-05-09 to 2023-01-15	2023-01-16 to 2023-12-17	2023-12-18 to 2024-09-21
Note: Predominance periods are defined as periods of 4 weeks or greater during which a subvariant maintained ≥50% prevalence based on data from www.covariants.org .					

Supplementary Figure 1:

Scatter plot of observed vs predicted rates of hospitalization per million population Denmark (A), The Netherlands (B) and the United States based on linear regression model #10 in Table 5. Dotted lines represent line y=x.

A.

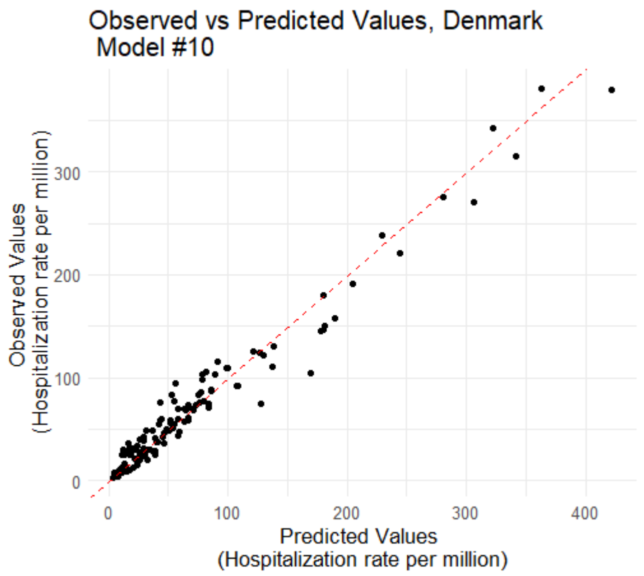

B.

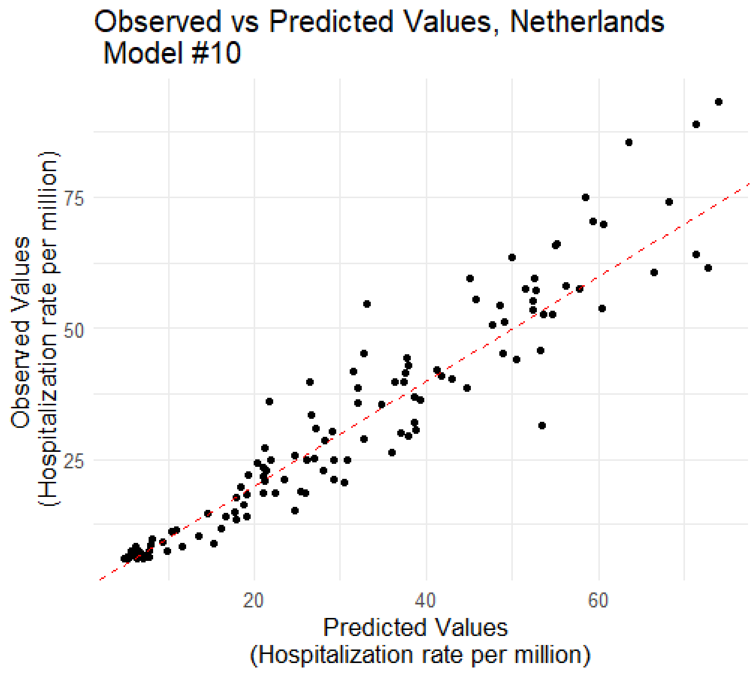

C.

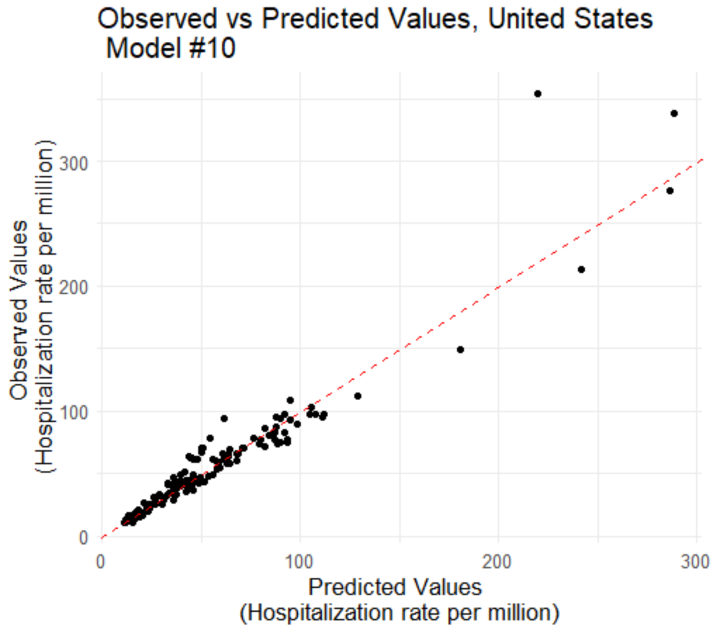
Observed vs Predicted Values, United States
Model #10
Observed Values
(Hospitalization rate per million)
0
100
200
300
Predicted Values
(Hospitalization rate per million)
0
100
200
300

Supplementary Figure 2:

Scatter plot of fitted values vs residuals in Denmark (A), The Netherlands (B) and the United States based on linear regression model #10 in Table 5. Dashed line represents y=0.

A.

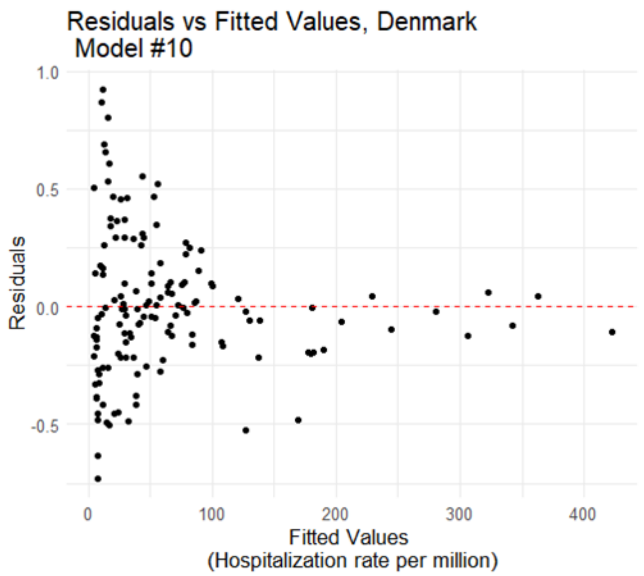

B.

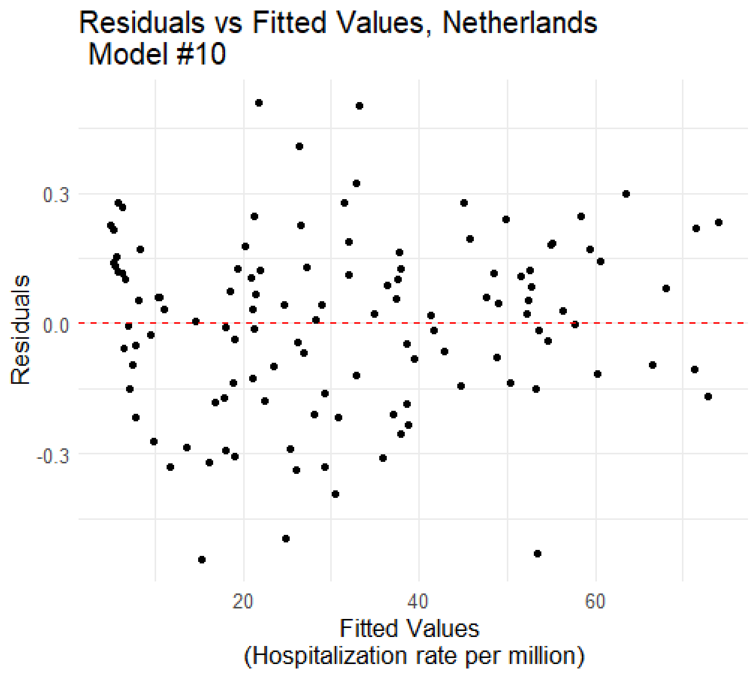

C.

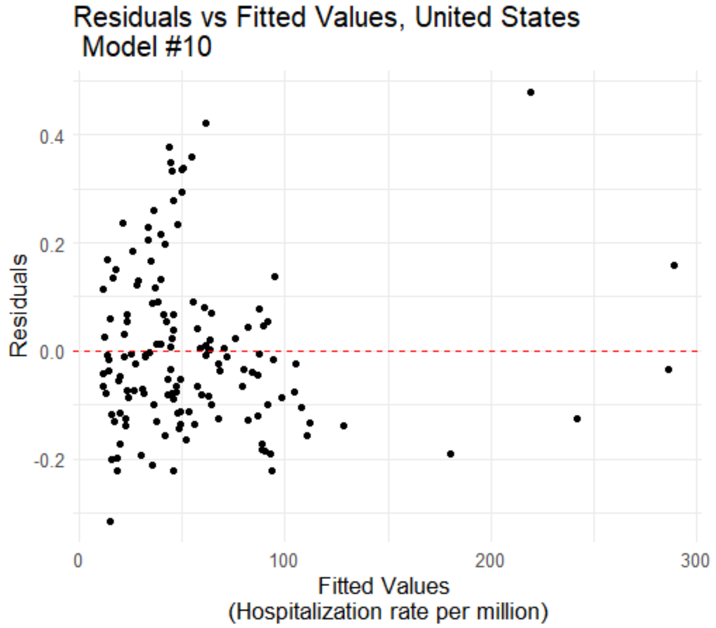

Residuals vs Fitted Values, United States
Model #10
Residuals
0.4
0.2
0.0
-0.2
0
100
200
300
Fitted Values
(Hospitalization rate per million)

Supplementary Figure 3:

Scatter plot of leverage vs studentized residuals in Denmark (A), The Netherlands (B) and the United States based on linear regression model #10 in Table 5. Dashed lines represent y = 3 and y = -3.

A.

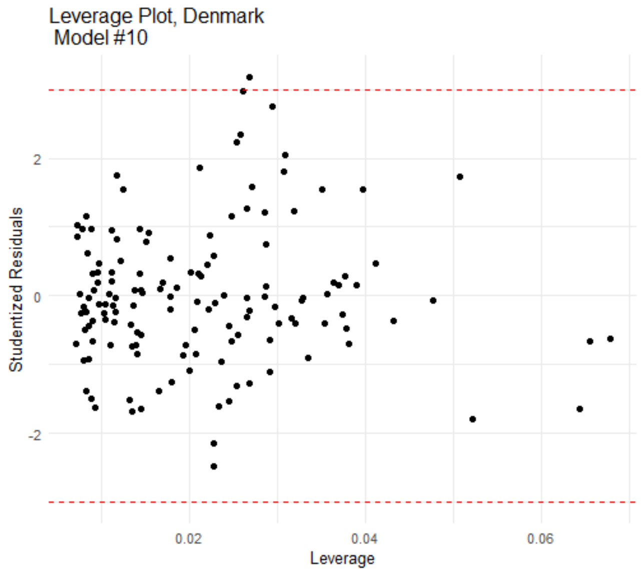

B.

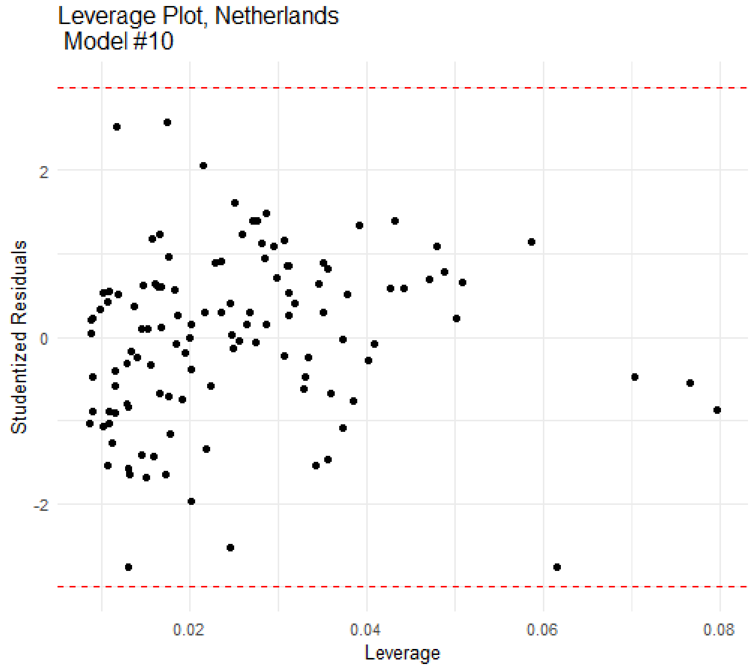

C.

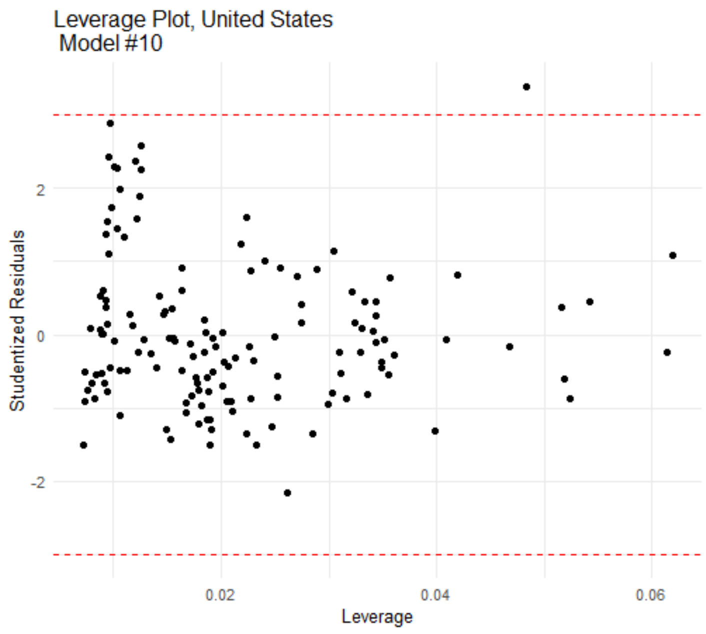
Leverage Plot, United States
Model #10
Studentized Residuals
2
0
-2
0.02
0.04
0.06
Leverage
